# *Staphylococcus epidermidis* Boosts Innate Immune Response by Activation of Gamma Delta T Cells and Induction of Perforin-2 in Human Skin

**DOI:** 10.3389/fimmu.2020.550946

**Published:** 2020-09-16

**Authors:** Irena Pastar, Katelyn O’Neill, Laura Padula, Cheyanne R. Head, Jamie L. Burgess, Vivien Chen, Denisse Garcia, Olivera Stojadinovic, Suzanne Hower, Gregory V. Plano, Seth R. Thaller, Marjana Tomic-Canic, Natasa Strbo

**Affiliations:** ^1^Wound Healing and Regenerative Medicine Research Program, Dr. Phillip Frost Department of Dermatology and Cutaneous Surgery, University of Miami Miller School of Medicine, Miami, FL, United States; ^2^Department of Microbiology and Immunology, University of Miami Miller School of Medicine, Miami, FL, United States; ^3^Division of Plastic Surgery Dewitt Daughtry, Department of Surgery, University of Miami Miller School of Medicine, Miami, FL, United States

**Keywords:** perforin-2/mpeg-1, human skin, innate immunity, *Staphylococcus epidermidis*, gamma delta T cells, cytotoxicity

## Abstract

Perforin-2 (P-2) is an antimicrobial protein with unique properties to kill intracellular bacteria. Gamma delta (GD) T cells, as the major T cell population in epithelial tissues, play a central role in protective and pathogenic immune responses in the skin. However, the tissue-specific mechanisms that control the innate immune response and the effector functions of GD T cells, especially the cross-talk with commensal organisms, are not very well understood. We hypothesized that the most prevalent skin commensal microorganism, *Staphylococcus epidermidis*, may play a role in regulating GD T cell-mediated cutaneous responses. We analyzed antimicrobial protein P-2 expression in human skin at a single cell resolution using an amplified fluorescence *in situ* hybridization approach to detect P-2 mRNA in combination with immunophenotyping. We show that *S. epidermidis* activates GD T cells and upregulates P-2 in human skin *ex vivo* in a cell-specific manner. Furthermore, P-2 upregulation following *S. epidermidis* stimulation correlates with increased ability of skin cells to kill intracellular *Staphylococcus aureus*. Our findings are the first to reveal that skin commensal bacteria induce P-2 expression, which may be utilized beneficially to modulate host innate immune responses and protect from skin infections.

## Introduction

Skin, in the same fashion as all other epithelial barrier sites (gastrointestinal, reproductive, and respiratory tracts) harbors a distinct community of commensal microbes that modulate the host immune system (1–3). One of the most common members of the healthy cutaneous microbiome is *Staphylococcus epidermidis*. *S. epidermidis* stimulates antimicrobial peptide production by skin cells (4–11), which may provide protection against pathogenic bacteria (4, 5, 10–12). Recent studies reported that colonization of mouse skin with *S. epidermidis* induced commensal-specific tissue (skin)-resident memory T cells that demonstrated immunoregulatory and tissue repair properties. This was proposed as a novel *S. epidermidis* mediated mechanism for rapid immune response and tissue protection from invasive pathogens (13–15).

Multiple lines of evidence have shown that gamma delta (GD) T cells display strong activities against bacteria (16–20), parasites (21), and viruses (22, 23). In marked contrast to αβ T lymphocytes (24–29), GD T cells recognize antigens independently of peptide processing and major histocompatibility complex (MHC)-restricted antigen presentation. They are activated by signs of tissue stress, including infected or transformed cells, and respond by deploying an immediate and efficient killing response or by regulating the immune response against them. Phosphoantigens and several other molecules of microbial origin have been proposed as GD T cell antigens accounting for the specific recognition of infected cells. These candidates include the *Staphylococcus aureus* superantigens Staphylococcal enterotoxin A (SEA) (and to a lesser extent staphylococcal enterotoxin E (SEE) (30, 31), which are recognized by the GD T cell receptor (TCR) independently from antigen processing and MHC presentation. Although GD T cells are one of the predominant lymphocyte subsets in mouse and human skin (32) that are essential for skin homeostatic and protective pathways against *S. aureus* (33), the contribution of commensal-derived antigens to the activation of GD T cells and their effector function, particularly their cytotoxic potential, has not been established. Furthermore, the extent to which GD T cells promote cutaneous tissue physiology remains to be determined.

Perforin-2 (P-2)/MPEG1 is a highly conserved member of the membrane attack complex (MAC)/perforin-like (PF)/cholesterol-dependent cytolysin (MACPF/CDC) superfamily (34–36). In contrast to all other MACPF/CDC members, P-2 is a type-1 transmembrane protein that traffics throughout the endosomal pathway to the late-endosome and phagosome (37–39). Therefore, P-2 can form pores in bacterial membranes and damage engulfed microbes within the phagolysosome (37, 40). In the absence of P-2, the other innate defense effectors including reactive oxygen species and nitric oxide, were unable to prevent the replication and systemic dissemination of intracellular pathogens (37, 41, 42). Dr. Eckhard Podack’s group was the first to report about major P-2 functions as an antibacterial effector protein of the innate immune system in phagocytic and in tissue forming cells (37, 41). Although we recently reported specific distribution of P-2 in normal human skin (43), the mechanisms involved in the regulation of P-2 expression have not been well established. Moreover, the effect of P-2 function within the complex system of host-microbe interactions has important implication for our understanding of skin immunity and diseases.

Here we established a human skin *ex vivo* model to study the effect of *S. epidermidis* on the skin innate immune response and on the novel antimicrobial protein P-2. We report that *S. epidermidis* activates skin GD T cells, specifically through P-2 induction, which has demonstrated antibacterial effects in other cell subsets (macrophages and fibroblasts) (37, 42). Importantly, *S. epidermidis* mediated induction of P-2 correlated with an enhanced ability of the skin cells to eliminate intracellular *S. aureus*.

## Materials and Methods

### Bacterial Strains and Culture Conditions

*Staphylococcus epidermidis* CCN021 and CCN0024, human commensal *S. epidermidis* strains, were obtained from GP (University of Miami). *S. epidermidis* ATCC 12228 was a gift from Prof. Davis (University of Miami). *S. epidermidis* CCN021 and CCN0024 were isolated from a healthy volunteer and characterized by phenotypic and qPCR identification techniques (44, 45). Staphylococci were routinely grown aerobically with agitation, at 37°C, in Luria-Bertani (LB) broth. For pre-treatment, bacteria were diluted in fresh LB and grown to mid-log growth phase. Before application on *ex vivo* human skin or single cell suspension, bacteria were harvested by centrifugation and washed with phosphate buffered saline (PBS). The bacterial density and the absence of contamination were controlled by numeration of colony forming units (CFU).

The GFP containing USA300 Methicillin resistant *Staphylococcus aureus* (MRSA) strain AH1726 [MRSA LAC (AH1263) + pCM29 (CmR)] (46) was obtained from GP (University of Miami). MRSA was grown aerobically with agitation overnight at 37°C in LB supplemented with 10 μg/mL chloramphenicol to retain the GFP plasmid.

### *Ex vivo* Human Skin Explant System

Discarded human skin tissue was obtained from voluntary reduction surgeries (*n* = 6) at the University of Miami (UM) Hospital and as such were found to be exempt from human subject research under CFR46.101.2 by the Institutional Review Board at the UM Miller School of Medicine.

Skin samples were processed to remove subcutaneous fat and washed with PBS. Multiple 8 mm punch biopsies were obtained from each specimen and placed individually into 0.4 μm PET-membrane *trans-*wells (Millipore) in a 12 well plate containing 1 ml media per well (RPMI, 10% FBS, 1% HEPES). Skin specimens were maintained at the air-liquid interface as previously described (43, 47–50).

### Human Skin Single Cell Suspension

Cells were isolated from healthy human skin using the MACS Whole Skin Dissociation Kit (Miltenyi 130-101-540). Briefly, subcutaneous fat was removed, and sterilization of human skin was optimized to remove any commensal or pathogenic microorganisms. Skin was washed with Gibco^®^ Antibiotic-Antimycotic (ABAM) (Life Technologies) to prevent bacterial and fungal contamination. This solution contains 10,000 units/mL of penicillin, 10,000 μg/mL of streptomycin, and 25 μg/mL of Gibco Amphotericin B. After washings with ABAM, skin was washed in PBS (46). Three 4 mm diameter punches were digested overnight at 37°C using enzymes from a whole-skin dissociation kit (Miltenyi, Bergisch Gladbach, Germany). The resulting cell suspension was filtered through a 70 μm cell strainer and centrifuged at 1,500 r.p.m. for 10 min at 4°C. The supernatant was removed, and the pellet was washed once with PBS. Obtained cell suspensions were washed with IMDM (Gibco-Thermo Fisher Scientific) supplemented with 10% heat-inactivated FBS, 2 mM L-glutamine, 0.15% sodium hydrogencarbonate, 1 mM sodium pyruvate, and non-essential amino acids.

### *Ex vivo* and *in vitro* Skin Stimulation With *S. epidermidis*

*Staphylococcus epidermidis* CCN021 was prepared for stimulation experiments as described above and 20 μL of the bacteria solution (approx. 6 Log CFU) was added centrally onto the epidermis while control samples were treated with PBS. After 24, 48, 72, and 96 h of incubation at 37°C in a 5% CO2 atmosphere, tissue samples were either digested with collagenase for further cell viability and FISH/Flow analysis (Supplementary Figure S2), preserved in RNA-later for RNA isolation, or used for CFU enumeration. CFU count was determined after overnight colony growth and expressed as CFU/ml.

Skin cell suspension obtained after whole-skin dissociation was used for *in vitro* stimulation with *S. epidermidis* CCN021, CCN0024, and ATCC 12228. Cells were plated on 24 well plates with 1 million cells per well and treated with *S. epidermidis* at a multiplicity of infection (MOI) of 20 for 24 h. The control cells were exposed to media only.

### Intracellular MRSA Killing Assay

After 24 h stimulation with *S. epidermidis*, skin cells were washed twice with warmed plain IMDM and infected with MRSA at an MOI of 20 for 1 h. Cells were washed twice with IMDM after infection and fresh media containing gentamicin (50 μg/mL) was added for 30 min to eliminate extracellular bacteria. Samples were collected 30 and 90 min after intracellular infection for enumeration of intracellular colony forming units (CFU) and for FISH flow analysis as described before (43). To release intracellular bacterial load, cells were subjected to hypotonic lysis with 0.1% Triton X in PBS. Lysates were plated on agar plates containing 10 μg/mL chloramphenicol for CFU quantification (43).

### FISH-Flow P-2 RNA Assay and Flow Cytometric Analysis

Single cell suspensions obtained from full thickness samples or after stimulation with *S. epidermidis* and MRSA were first labeled with live/dead detection kit (Yellow Amine, Thermo Fisher Scientific) and then with the following fluorescently labeled antibodies: CD45-Alexa Fluor 700, TCR GD-PE-Cy7, CD31-PacBlue, CD104-FITC, CD325-PerCPCy5.5, and CCRL1-PE (Biolegend, San Diego, CA, United States). We also stained cells with fluorescently labeled antibodies for TLR1-BV570, TLR2-PE, TLR6-BV605, and TCR GD 1 FITC (Biolegend, San Diego, CA, United States). P-2 mRNA was detected using an amplified signal FISH technique (PrimeFlow; Affymetrix/eBioscience-Thermo Fisher Scientific). For mRNA detection, target probe hybridization was performed using type 1 (AlexaFluor647) probes for P-2 as described (43). Approximately 20,000 cell events were acquired from each sample on flow cytometer equipped with 405 nm, 488 nm, 642 nm, and 785 nm (SSC) lasers (Fortessa X-50, BD Immunocytometry Systems, San Jose, CA, United States). Spectral compensation was completed using single color control samples and antibody capture beads (BD Biosciences). Data were analyzed using FlowJo version 10.2 (TreeStar).

### GD T Cell Sorting and Real-Time PCR

Two-way sorting was performed to obtain purified GD T cells by sterile sorting on a SONY SH800S cell sorter (SONY Biotechnology, San Jose, CA, United States). Briefly, single cell suspensions were labeled with Live/Dead Violet, CD45, CD3, and TCR GD. GD T cells were sorted as Live/Dead-CD45+ CD3+ TCR GD+ cell population. 5,000–10,000 sorted cells were collected from three donors. Purity of sorted GD T population was >97%.

After sorting, cells were stimulated with *S. epidermidis* at an MOI of 20 for 1 h, washed with PBS, spun down, and kept on ice briefly prior to performing one-step reverse transcription and cDNA amplification of specific targets using a pool of TaqmanTM gene expression assays (Thermo Fisher Scientific). Resulting cDNA was loaded onto BioMark IFC 96 × 96 chip (Fluidigm) according to the manufacturer’s protocol. Raw data underwent “cellular detection rate” (CDR) filtering to remove outlier samples and genes based on dataset distribution (51, 52). CD74 (also known as HLADG) was used as a surrogate for the presence of a cell (i.e., loading control) due to its stable expression in lymphocytes. Cells that had low or absent CD74 expression exhibited reduced gene expression globally and were removed from analysis. Differential gene expression analysis was subsequently performed to contrast transcriptional profiles of GD T cells between unstimulated and *S. epidermidis* stimulated samples.

### RT-PCR for Antimicrobial Peptides and Pro-inflammatory Cytokines

Total RNA from human skin was extracted using the miRNeasy kit (QIAGEN, Valencia, CA, United States) per manufacturer’s instructions as previously described (49). cDNA was made with qScript^TM^ Synthesis kit (Quanta BioSciences Inc.,Gaithersburg, MD, United States). ARPC2 was used as a reference gene for normalization, forward 5′-TCCGGGACTACCTGCACTAC-3′, reverse 5′-GGTTCAGCACCTTGAGGAAG-3′. All real-time PCR (qPCR) reactions were performed in triplicate using PerfeCTa^®^ SYBR^®^ Green SuperMix (Quanta BioSciences) and quantified using the ddCT method. The primer sequences were IL-1α forward 5′-AGATGCCTGAGATACCCAAAACC-3′ reverse 5′-CCAAGCACACCCAGTAGTCT-3′, defensin β4 (DefB4) forward 5′-GGTGGTATAGGCGATCCTGTT-3′ reverse 5′-AGGGCAAAAGACTGGATGACA-3′, and cathelicidin (LL37) forward 5′-GGGCAAAAGACTGGATGACA-3′ reverse 5′-TCTTGAAGTCACAATCCTCTGGT-3′.

### Statistical Analysis

All experiments were conducted independently at least three times on different days. Comparisons of flow cytometry cell frequencies was measured by the two-way ANOVA test with Holm-Sidak multiple-comparison test, ^∗^*p* < 0.05, ^∗∗^*p* < 0.01, and ^∗∗∗^*p* < 0.001 or Student *t*-test using the Prism software (GraphPad software). Comparisons of PCR array data were performed using *t*-test (two tail distribution and equal variances between the two groups) based on the triplicate 2^(−ΔCT) values for each gene in the *S. epidermidis* treated group and control group. Error bars in all figures are reported as a SEM.

## Results

### *S. epidermidis* Contributes to Increased Number of Human GD T Cells

**FIGURE 1:**
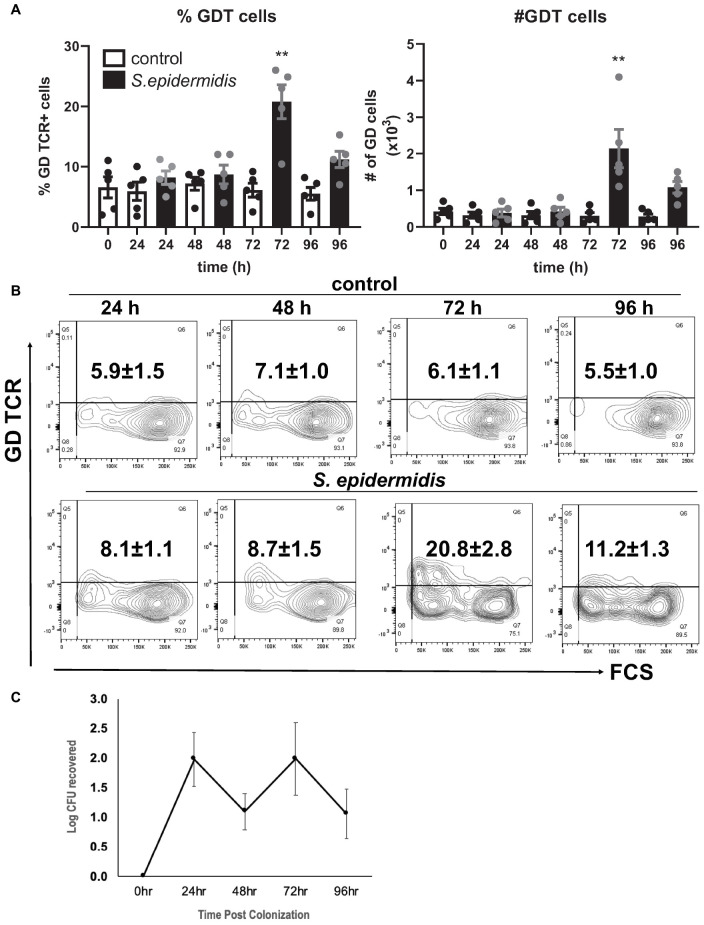
*Staphylococcus epidermidis* increases the number of GD T cells in human skin *ex vivo*. Control, uncolonized, and *S. epidermidis* colonized skin was maintained on air liquid interface and collected at indicated time points (0, 24, 48, 72, and 96 h). Single cell suspensions were obtained and labeled with live/dead stain, CD45, CD3, and GD TCR. **(A)** Cells were analyzed using flow cytometry and gated on the CD45+ CD3+ GDT+ population. Bar graphs show SEM frequency (%) and SEM number (#) of skin GD T cells (*n* = 5). **(B)** Representative contour plots showing frequency of GD TCR in control and *S. epidermidis* colonized skin. **(C)** Number of *S. epidermidis* colony forming units (CFU) recovered from *ex vivo* skin explants colonized with *S. epidermidis* CCN021 on day 0 through day 4. Data represent at least two technical replicates and five independent biological replicates per group. **p < 0.01 (two-way ANOVA with Holm-Sidak multiple-comparison test).

*Staphylococcus epidermidis* is an important skin commensal organism and modulator of cutaneous innate immune responses (9, 10). Here we established an *ex vivo* skin model of *S. epidermidis* colonization to study the effect of this commensal microorganism on skin innate immune responses including GD T cell activity. *S. epidermidis* was topically applied onto the epidermis. Tissue was collected at different time points during colonization and then dissociated into single cell suspensions (Figure 1). There were no statistical differences in viability of *S. epidermidis* treated and control tissue (Supplementary Figure S1). We analyzed the GD T cell subset in the control and *S. epidermidis* colonized human skin by flow cytometry and observed a statistically significant (*p* < 0.01) increase in the frequency as well as in the total number of GD T cells within live, CD45+ CD3+ skin cells after 72 h of *S. epidermidis* stimulation compared to control tissue (Figures 1A,B). We confirmed *S. epidermidis* colonization in human skin by CFU quantification (Figure 1C).

### *S. epidermidis* Induces P-2 in Human GD T Cells, Keratinocytes, and Papillary Fibroblasts *ex vivo*

**FIGURE 2:**
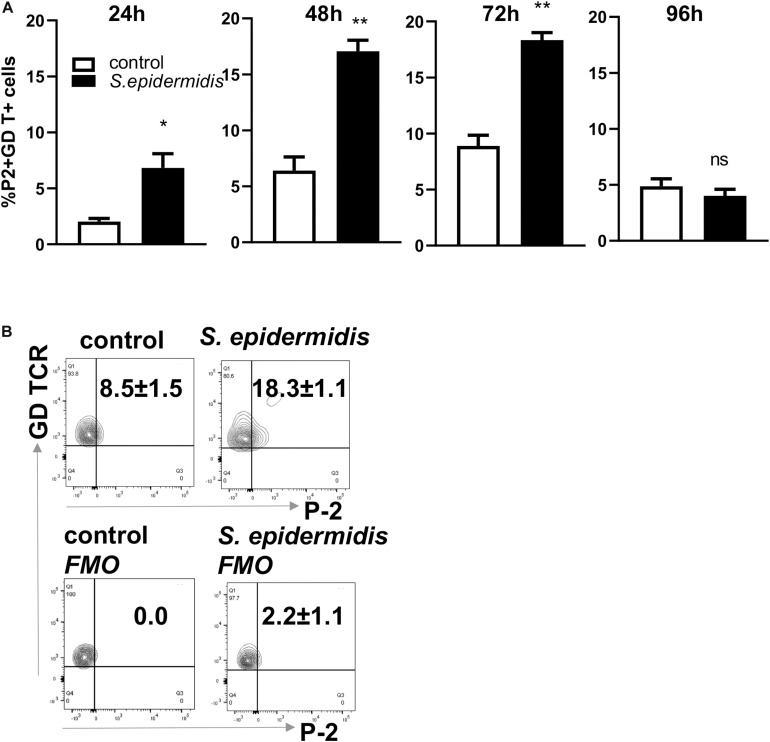
Colonization of human skin with *Staphylococcus epidermis* induces P-2 in GD T cells. **(A)** Control and *S. epidermidis* colonized human skin was collected at indicated time points (24, 48, 72, and 96 h) for FISH-Flow. Single cell suspensions were obtained using Collagenase D and labeled with live/dead stain. Using FISH-Flow RNA assay, P-2 RNA levels were analyzed in the CD45+, CD3+ GD TCR+ cell population. **(B)** Representative dot plot graph showing expression of mRNA P-2 in gated CD45+ CD3+ GD TCR+ T cells at 72 h in *S. epidermidis* colonized skin or non-colonized (control). FMO-fluorescence minus one. Bar graphs show SEM of P-2 mRNA positive cells within skin GD T cells (*n* = 3). Data represent at least two technical replicates with three independent biological replicates per group. *p < 0.05, **p < 0.01 as calculated using Student *t*-test.

**FIGURE 3:**
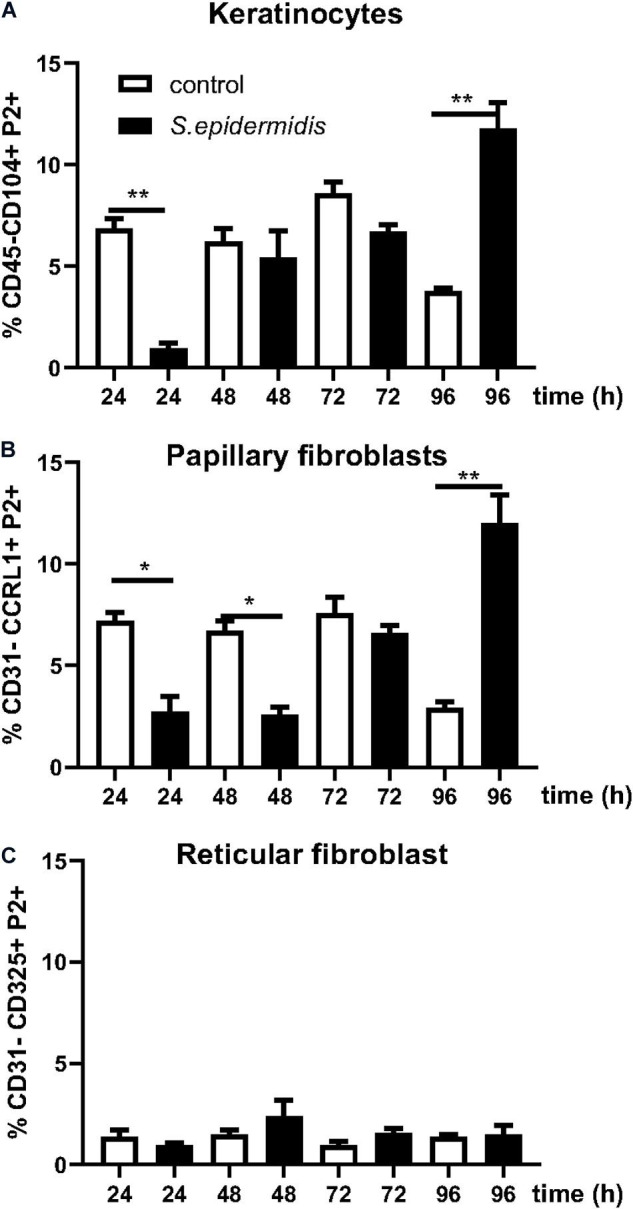
*Staphylococcus epidermidis* colonization induces P-2 in human keratinocytes and papillary fibroblasts. Control, non-colonized, and *S. epidermidis* colonized human skin was maintained at air liquid interface and collected at indicated time points (24, 48, 72, and 96 h) for FISH-Flow. Single cell suspensions were labeled with live/dead stain. Using FISH-Flow RNA assay, P-2 RNA levels were analyzed in **(A)** CD45-, CD31-, CD104+ cells (keratinocytes), or **(B)** CD45-, CD31-, CCRL1+ (papillary fibroblasts), and **(C)** CD45-, CD31-, CD325+ cells (reticular fibroblasts). Bar graphs show percentage of P-2 mRNA positive cells within each CD45-CD31-skin cell population. Data represent at least two experiments with three independent biological replicates per group. *p < 0.05, **p < 0.01 (two-way ANOVA with Holm-Sidak multiple-comparison test).

We have previously described an amplified fluorescence *in situ* hybridization (FISH) technique for detection of mRNA in combination with immune-phenotyping in human skin (43). We and others found that P-2 is an antimicrobial protein crucial for intracellular bacteria killing (37, 38, 40, 41). Here, we analyzed P-2 expression in different skin cell subsets after stimulation with *S. epidermidis*. First, we found that P-2 expression was significantly upregulated (*p* < 0.05 and *p* < 0.01) in GD T cells from human skin explants colonized with *S. epidermidis* at 24, 48, and 72 h compared to the uncolonized control (Figures 2A,B). Moreover, our analysis revealed that *S. epidermidis* stimulation for 96 h upregulated P-2 in the basal layer keratinocytes after an initial suppression observed at 24 h (CD45-CD31-CD104+ cells) (Figure 3). Two major human skin fibroblast subsets, papillary and reticular fibroblasts, based on their expression of CCRL1 and CD325, respectively (53, 54) were also tested. We found that only papillary fibroblasts, CCRL1+ cells, upregulate P-2 96 h post *S. epidermidis* colonization. P-2 expression in reticular fibroblasts was not affected at any time point and was lower overall compared to other cell subtypes (Figure 3).

### Antimicrobial Peptides Are Upregulated in Human Skin by *S. epidermidis*

**FIGURE 4:**
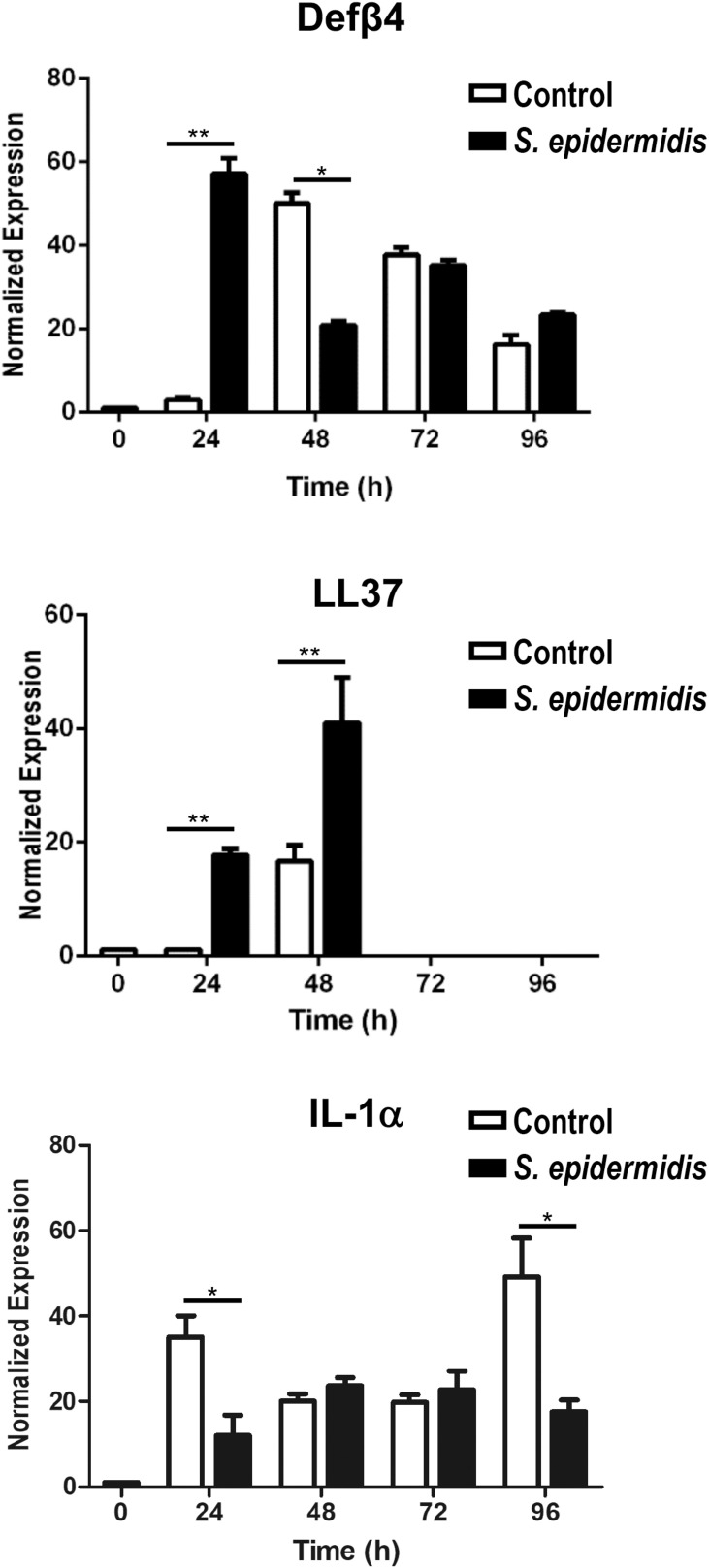
Antimicrobial and inflammatory responses mediated by *Staphylococcus epidermidis* in human *ex vivo* model. Expression levels of antimicrobial peptides Defβ4, LL-37, and pro-inflammatory cytokine IL-1α were evaluated by qPCR from non-colonized and *S. epidermidis* colonized skin at 24, 48, 72, and 96 h (*n* = 3 for each treatment group and time point). *p < 0.05, **p < 0.01 as calculated using Student *t*-test.

*Staphylococcus epidermidis* isolates from healthy adults have been reported to show widespread production of bacteriocins (55) and in addition they can stimulate keratinocytes to produce antimicrobial peptides (4). We investigated if *S. epidermidis* triggers expression of antimicrobial peptides in our *ex vivo* skin model. We found that 24 h of *S. epidermidis* colonization significantly induced expression of defensin β4 (Defβ4) and cathelicidin (LL37) (*p* < 0.01) (Figure 4). Upregulation of LL37 was maintained 48 h post *S. epidermidis* colonization (*p* < 0.01) in contrast to defensin Defβ4 that was downregulated (*p* < 0.05). In addition, *S. epidermidis* colonization of human skin resulted in downregulation of pro-inflammatory IL-1α after 24 and 96 h (*p* < 0.05) (Figure 4).

### Early Regulation of GD T Cell Gene Expression by *S. epidermidis*

**FIGURE 5:**
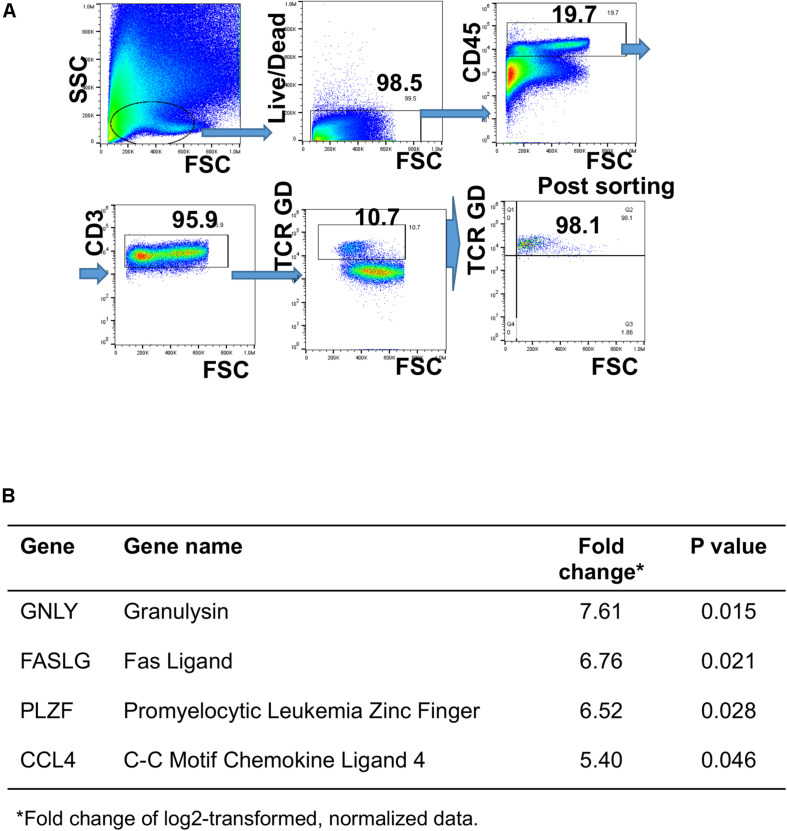
Differential gene expression in human GD T cells as an early response to *Staphylococcus epidermidis*. Human skin cells were isolated using the Miltenyi Whole Skin Dissociation kit (Miltenyi, Bergisch-Gladbach, Germany) and GD T cells were sorted from the skin cell suspension using fluorescence-activated cell sorting. **(A)** Gating strategy for GD T cell sorting. **(B)** Cells were stimulated with *S. epidermidis* for 1 h and changes in gene expression between uninfected and infected cells were measured using the BioMark IFC 96 × 96 chip (Fluidigm). Changes in gene expression are expressed as log2 of fold change (*n* = 3). *P*-values were calculated using Student *t*-test.

Human GD T cells in the skin exhibit both pro-inflammatory and regulatory functions (32). Deciphering the underlying mechanisms that contribute to induction of effector vs. regulatory GD T cell functions, including expression of cytotoxic molecules, is key to understanding skin homeostasis. To understand the effect of *S. epidermidis* on skin GD T cells during initial phases of colonization, we sorted GD T cells from normal skin (Figure 5A) and stimulated them with *S. epidermidis for* 1 h. We evaluated the expression of well-known genes previously described to play a role in GD T cell cytotoxic functions. We found a 6-7-fold induction in Fas ligand (FASLG) and Granulysin (GNLY) in *S. epidermidis* treated cells compared to control, untreated GD T cells (Figure 5B). Additionally, we observed increased expression of the transcription factor PLZF, which is responsible for selection of GD innate natural killer T cells (56), as well as CCL4, a monokine with inflammatory and chemokinetic properties (Figure 5B). Previous studies have observed increased CCL4 expression by GD T cells following engagement of the natural cytotoxicity receptor NKp30 on the GD T cell surface (57).

### Intracellular MRSA Killing Is Enhanced After Exposure to *S. epidermidis*

**FIGURE 6:**
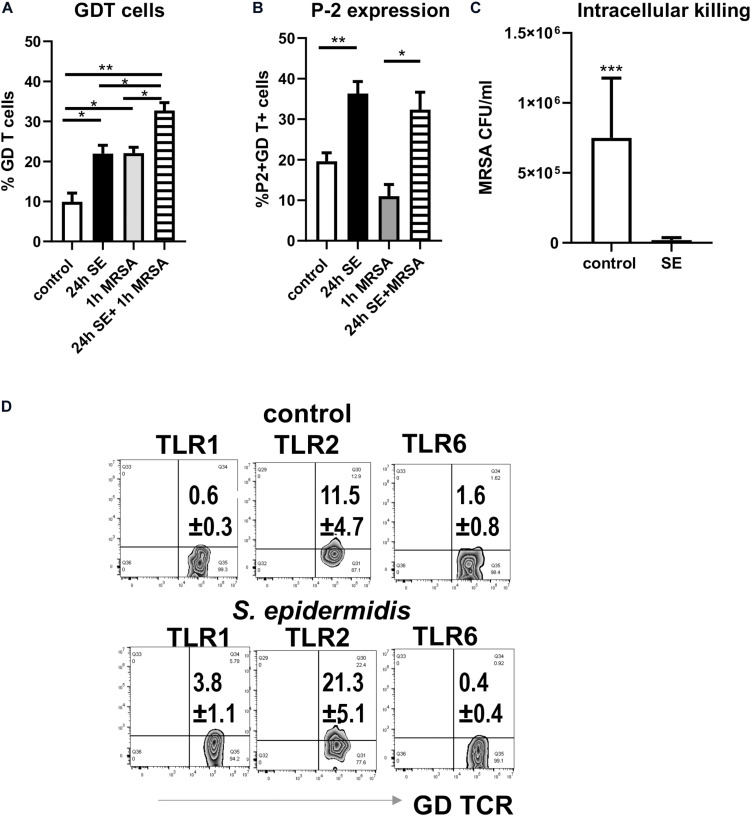
Pre-treatment of skin cells with *Staphylococcus epidermidis* increases frequency of GD T cells, stimulates P-2 expression, and limits survival of intracellular MRSA. Single skin cells were exposed to *S. epidermidis* at MOI 1:20 or media control for 24 h. After washing to remove *S. epidermidis*, cells were infected with MRSA (MOI 1:20) for 1 h to allow intracellular infection, and extracellular bacteria were subsequently removed by gentamicin treatment. **(A)** Frequency of GDT cells and **(B)** P2 mRNA expression in CD45+ CD3+ GD TCR+ cells as determined by FISH-Flow (*n* = 3 biological replicates). **(C)** Bar graph showing the number of intracellular MRSA (CFU/ml) upon hypotonic lysis of control and *S. epidermidis* pre-treated cells (*n* = 3 biological replicates). **(D)** Expression of TLR1, TLR2, and TLR6 on gated CD45+ CD3+ TCR GD+ T cells. Data represent at least two experiments with three independent biological replicates per group. *p < 0.05, **p < 0.01 as calculated using two-way ANOVA with Holm-Sidak multiple-comparison **(A)** and ***p < 0.001 as calculated using Student *t*-test **(B)**.

We have previously reported that MRSA, the most common cutaneous pathogen, suppresses P-2 induction in skin cells (43), revealing a novel mechanism by which *S. aureus* may escape cutaneous immunity to cause persistent infections. Here, we report that in contrast to MRSA (43), *S. epidermidis* up-regulates P-2 expression in an *ex vivo* skin model (see Figure 1) and in the single cell suspension culture model (Figure 6). In order to further analyze *S. epidermidis*-mediated induction of P-2 in *ex vivo* human skin, we isolated skin cells and established a single cell type culture system. We found an increase in the frequency of GD T cells after 24 h stimulation with *S. epidermidis* (Figure 6A), which agrees with findings from the *ex vivo* human skin model (Figure 1). Furthermore, expression of P-2 was also increased in the GD T cells after 24 h of *S. epidermidis* stimulation (Figure 6B). Most importantly, we show that cells stimulated with *S. epidermidis* demonstrate an increased capability to kill intracellular MRSA (Figure 6C). We observed this same result after repeating stimulations with 2 additional *S. epidermidis* strains, *S. epidermidis* ATCC 12228 and commensal isolate *S. epidermidis* CCN0024 (Supplementary Figure S2). In addition, we found that *S. epidermidis* CCN021 stimulated GD T cells upregulate expression of TLR2 and TLR1, but not TLR6 (Figure 6D).

## Discussion

Pore-forming proteins permeabilize membranes of infected cells targeted for immune elimination and together with antimicrobial peptides represent the key effector molecules of the epithelial barriers. GD T cells, as surveillance cells in the skin, constitutively express mRNA for granzyme A and B and perforin and contain significant esterase activity (58). We provided the first evidence to show that human skin GD T cells constitutively express antimicrobial protein P-2 (43). In contrast to other secreted pore forming proteins, P-2 is a transmembrane protein that efficiently kills intracellular bacteria. IFNγ, type I interferons, and LPS have been implicated in the regulation of its expression (37, 38). We have previously reported that MRSA, the most common skin pathogen, suppresses P2-induction in skin cells (43), revealing a novel mechanism by which *S. aureus* may escape cutaneous immunity to cause persistent infections. Here we show that, in contrast to MRSA, *S. epidermidis* up-regulates P-2 expression in human skin and in single cell suspensions. We also demonstrate that *S. epidermidis* upregulates P-2 mRNA expression in multiple skin cell types including GD T cells, basal keratinocytes, and papillary fibroblasts. Most importantly, we observed a decrease in number of intracellular MRSA in skin stimulated by *S. epidermidis*, which correlates with *S. epidermidis*-mediated P-2 induction. These data provide new insights regarding mechanisms of P-2 expression and function and elucidate novel approaches to protect skin from infections caused by intracellular pathogens.

Gamma delta T cells represent a major T cell subset involved in the surveillance of epithelial surfaces (skin, gastrointestinal, reproductive, and respiratory tracts). It is well establish that GD T cells, upon recognition of pathogens, effectively proliferate, secrete pro-inflammatory cytokines, and activate their cytolytic machinery (perforin and granzymes) to kill the pathogen (59). Clonally expanded GD T cells can establish long-lasting immunity against recurrent *S. aureus* skin infections (33). In contrast, GD T cell deficient mice develop large skin lesions after infection with *S. aureus* (60). However, encounters with commensal microbes by skin GD T cells and how such interactions affect their response to pathogens remains poorly understood. Here we provide the first evidence that the common skin commensal, *S. epidermidis*, upregulates the frequency of GD T cells and induces the expression of P-2, which is associated with an increased capability to eliminate intracellular MRSA. Our data on increased frequency of skin GD T cells after colonization with *S. epidermidis* supports the hypothesis that under normal conditions the presence of *S. epidermidis* on the skin surface strengthens cutaneous innate defenses (9, 10).

We observed that during the early steps of colonization, prior to P-2 induction, *S. epidermidis* upregulates the GD T cell cytotoxic molecules Fas Ligand (FASLG) and granulysin (GNLY). This may contribute to the GD T cell mediated antimicrobial immune response, in addition to P-2 induction at later time points. We are currently expanding these studies to *in vivo* animal models. *S. epidermidis*, when topically applied to murine skin, induces specific IL-17 producing T cells that persist as tissue-resident memory T cells (13). However, to the best of our knowledge, our study is the first report that shows specific effect of *S. epidermidis* on induction of human skin GD T cell responses.

Previous reports indicate that a commensal strain of *S. epidermidis* and non-commensal strain *S. carnosus* have different modifications of the lipoprotein (Lpp) lipid moieties (61). The essential receptor for recognition of bacterial Lpp is TLR2. However, the degree of acylation at the lipid moiety can be discriminated by additional TLRs, such as TLR1 and TLR6, which form heterodimers with TLR2 (62–64). Importantly, these lipoprotein modifications were implicated in the differential immune responses where commensal staphylococcal species dampened IFNγ, TNFα, and IL-12 production compared to pathogenic staphylococcal species (61). We have found that a 24 h stimulation with *S. epidermidis* upregulates TLR2 and TLR1, but not TLR6 on GD T cells. We postulate that *S. epidermidis*, through recognition of TLR2/TLR1 heterodimers on cutaneous GD T cells, regulates not only Th1 responses but also cytotoxic mediators such as P-2. The recognition of TLR2/TLR1 heterodimers may even be strain specific (12) warranting further studies on the mechanisms of P-2 induction by *S. epidermidis* CCN021.

It has been shown that *S. epidermidis* colonization of skin induces AMP production by keratinocytes (5, 7, 8, 65). Our findings regarding induction of LL37 and Defβ4 during early phases of human skin colonization with *S. epidermidis* are in line with these results. Kinetics of P-2 induction upon *S. epidermidis* colonization shows dynamic control and cell specificity that integrates with the kinetics of AMP production. In GD T cells, the induction of P-2 is rapid and maintained, persisting from 24 to 72 h post colonization whereas in keratinocytes and papillary fibroblasts it shows complementary activation at 96 h. These data suggest that the initial protective response derives from GD T cells whereas in keratinocytes activation of P-2 follows initial activation of AMPs, cathelicidin, and β-defensin. The human skin *ex vivo* model is comprised of the epidermis and dermis with no circulation, thus limiting the studies on modulation of the immune response by *S. epidermidis* to resident innate immune cells, while the potential role of adaptive immunity would require *in vivo* models.

The initial findings presented here also provide a functional readout of *S. epidermidis* colonization and P-2 upregulation: decrease of the intracellular pathogen *S. aureu*s. We observed suppression of the pro-inflammatory cytokine IL-1α after colonization with *S. epidermidis.* Previously, we showed that *S. aureu*s induces IL-1α in non-healing diabetic foot ulcers (50), suggesting that *S. epidermidi*s may have additional mechanisms to neutralize the damaging effects of pathogenic organisms. The limitation of our study was sequential stimulation of human skin and primary cells by *S. epidermidis* and *S. aureus*. Future *in vivo* studies are required to confirm antimicrobial effects of *S. epidermidis* in the presence of pathogenic *S. aureus*. Additionally, future studies that block P-2 expression will be necessary to confirm that enhanced *S. aureus* killing upon *S. epidermidis* treatment is solely due to P-2 upregulation. Despite these limitations, this work provides an intriguing possibility that colonization of *S. epidermidis* may prevent and/or protect from bacterial skin infections through modulation of P-2.

In summary, we confirmed that colonization with commensal *S. epidermidis* in human *ex vivo* skin modulates the innate immune system by activating GD T cells, promoting antimicrobial peptide production, and upregulating the antimicrobial protein P-2. Understanding how commensal bacteria regulate P-2 expression represents the first step toward identifying mechanisms by which P-2 contributes to cutaneous homeostasis and host defense mechanisms and may reveal new approaches for preventing and treating skin infections.

## Data Availability Statement

The raw data supporting the conclusions of this article will be made available by the authors, without undue reservation.

## Ethics Statement

The studies involving human participants were reviewed and approved by Institutional Review Board at the UM Miller School of Medicine (exempt from human subject research under CFR46.101.2). The patients provided their written informed consent to participate in this study.

## Author Contributions

MT-C and NS obtained funding, and conceived and coordinated the experiments. IP, KO’N, LP, CH, JB, VC, DG, OS, and SH performed the experiments and analyzed the data. KO’N, IP, and NS performed the statistical analyses. GP and ST provided reagents. NS, IP, and MT-C wrote the manuscript. All authors were involved in writing and had final approval of the submitted and published versions of the manuscript.

## Conflict of Interest

The authors declare that the research was conducted in the absence of any commercial or financial relationships that could be construed as a potential conflict of interest.

## Supplementary Material

FIGURE S1 Cell viability of *ex vivo* skin tissue with or without *S. epidermidis* colonization via flow cytometry (data represented as mean ± SEM, *n* = 3–4 skin samples).

FIGURE S2 Pre-treatment of skin cells with *S. epidermidis* CCN0024 and ATCC 12228 strains limits intracellular MRSA survival. Single skin cells were exposed to single *S. epidermidis* strain at MOI 1:20 or media control for 24 h. After washing to remove *S. epidermidis*, cells were infected with MRSA (MOI 1:20) for 1 h to allow intracellular infection, and extracellular bacteria were subsequently removed by gentamicin treatment. Bar graph shows the number of intracellular MRSA (CFU/ml) upon hypotonic lysis of control and *S. epidermidis* pre-treated cells. ^****^*p* < 0.0001 as calculated using one-way ANOVA with Dunnett’s multiple comparisons test, which compared each *S. epidermidis* pre-treatment with non-pretreated control cells.
