# Corrigendum: *Staphylococcus epidermidis* Boosts Innate Immune Response by Activation of Gamma Delta T Cells and Induction of Perforin-2 in Human Skin

**DOI:** 10.3389/fimmu.2021.741437

**Published:** 2021-08-10

**Authors:** Irena Pastar, Katelyn O’Neill, Laura Padula, Cheyanne R. Head, Jamie L. Burgess, Vivien Chen, Denisse Garcia, Olivera Stojadinovic, Suzanne Hower, Gregory V. Plano, Seth R. Thaller, Marjana Tomic-Canic, Natasa Strbo

**Affiliations:** ^1^Wound Healing and Regenerative Medicine Research Program, Dr. Phillip Frost Department of Dermatology and Cutaneous Surgery, University of Miami Miller School of Medicine, Miami, FL, United States; ^2^Department of Microbiology and Immunology, University of Miami Miller School of Medicine, Miami, FL, United States; ^3^Division of Plastic Surgery Dewitt Daughtry Department of Surgery, University of Miami Miller School of Medicine, Miami, FL, United States

**Keywords:** perforin-2/mpeg-1, human skin, innate immunity, *Staphylococcus epidermidis*, gamma delta T cells, cytotoxicity

In the original article, there was a mistake in **Figure 1B** as published. The incorrect contour plots for the conditions Control 24 h and *S. epidermidis* 48 h were mistakenly included into **Figure 1B**. The correct representative contour plots in **Figure 1B** appear below.

**Figure 1:**
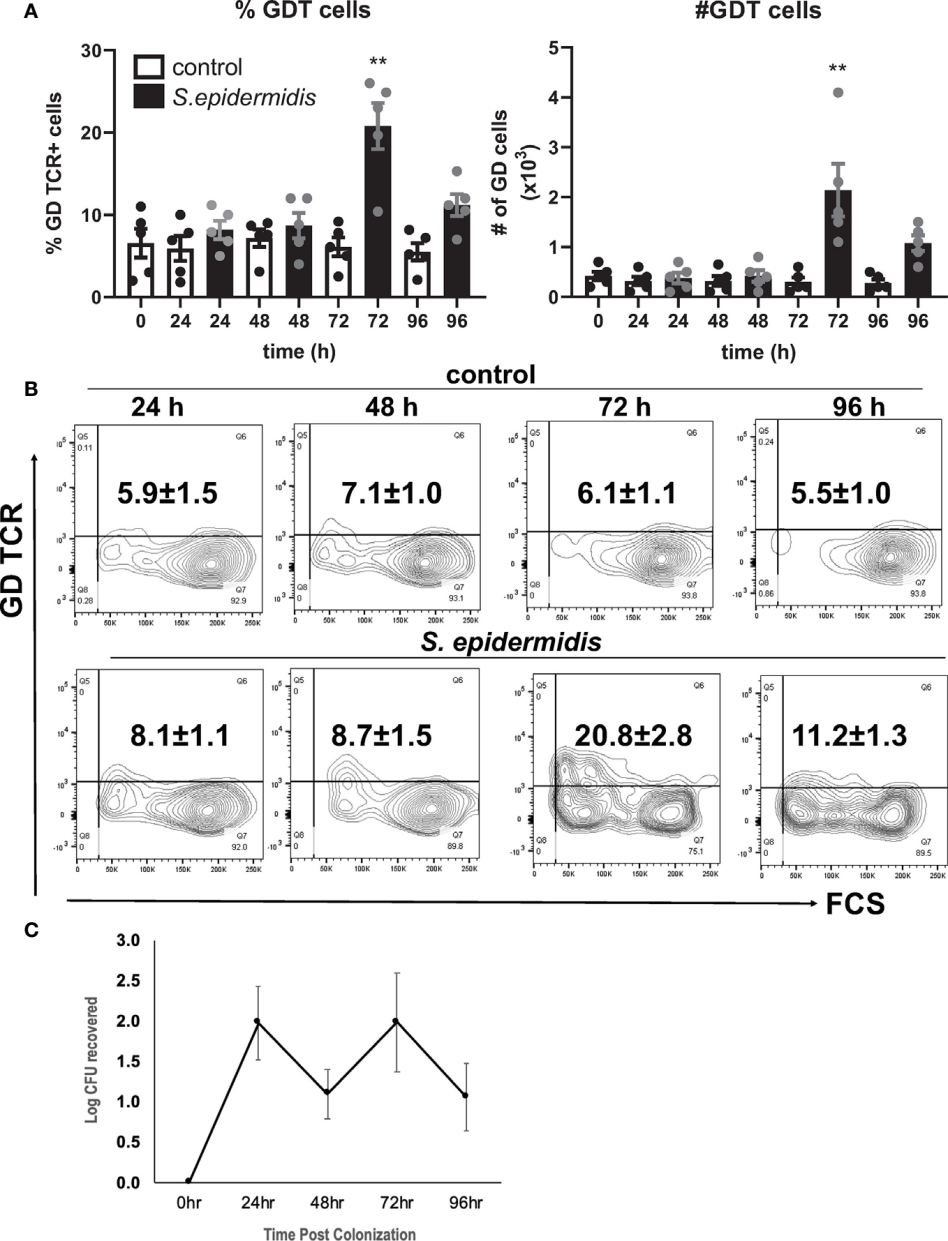
*Staphylococcus epidermidis* increases the number of GD T cells in human skin ex vivo. Control, uncolonized, and S. epidermidis colonized skin was maintained on air liquid interface and collected at indicated time points (0, 24, 48, 72, and 96 h). Single cell suspensions were obtained and labeled with live/dead stain, CD45, CD3, and GD TCR. **(A)** Cells were analyzed using flow cytometry and gated on the CD45+ CD3+ GDT+ population. Bar graphs show SEM frequency (%) and SEM number (#) of skin GD T cells (n = 5). **(B)** Representative contour plots showing frequency of GD TCR in control and S. epidermidis colonized skin. **(C)** Number of S. epidermidis colony forming units (CFU) recovered from ex vivo skin explants colonized with S. epidermidis CCN021 on day 0 through day 4. Data represent at least two technical replicates and five independent biological replicates per group. **p < 0.01 (two-way ANOVA with Holm-Sidak multiple-comparison test).

The authors apologize for this error and state that this does not change the scientific conclusions of the article in any way. The original article has been updated.

## Publisher’s Note

All claims expressed in this article are solely those of the authors and do not necessarily represent those of their affiliated organizations, or those of the publisher, the editors and the reviewers. Any product that may be evaluated in this article, or claim that may be made by its manufacturer, is not guaranteed or endorsed by the publisher.

