# Supplementary figures and images for: Staphylococcus epidermidis Boosts Innate Immune Response by Activation of Gamma Delta T Cells and Induction of Perforin-2 in Human Skin
Source: Front Immunol. 2020 Sep 16;11:550946. doi: 10.3389/fimmu.2020.550946 (PMC7525037; doi:10.3389/fimmu.2020.550946)

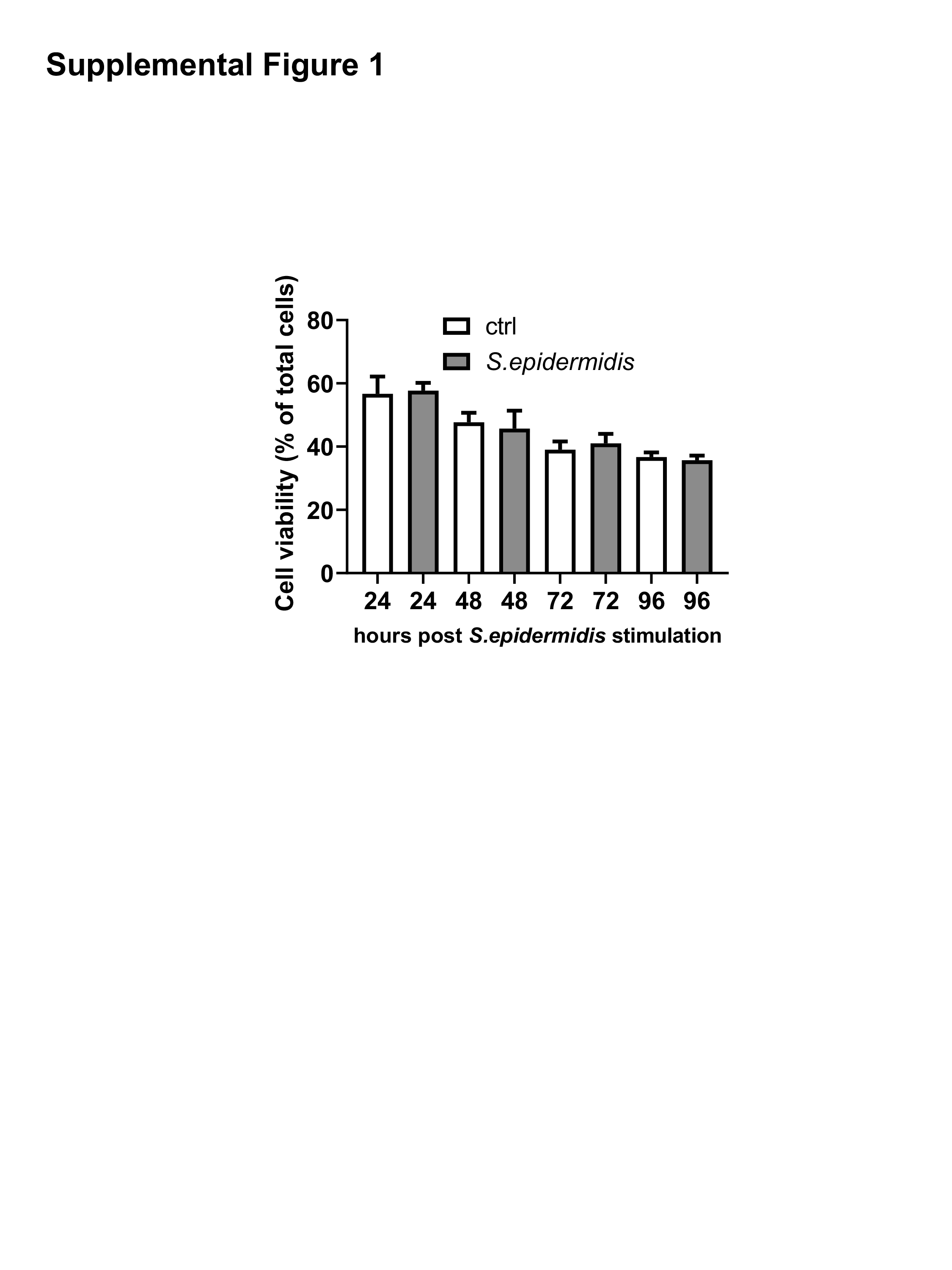

Supplement: FIGURE S1 — Cell viability of ex vivo skin tissue with or without S. epidermidis colonization via flow cytometry (data represented as mean ± SEM, n = 3–4 skin samples). [file Image_1.tif]

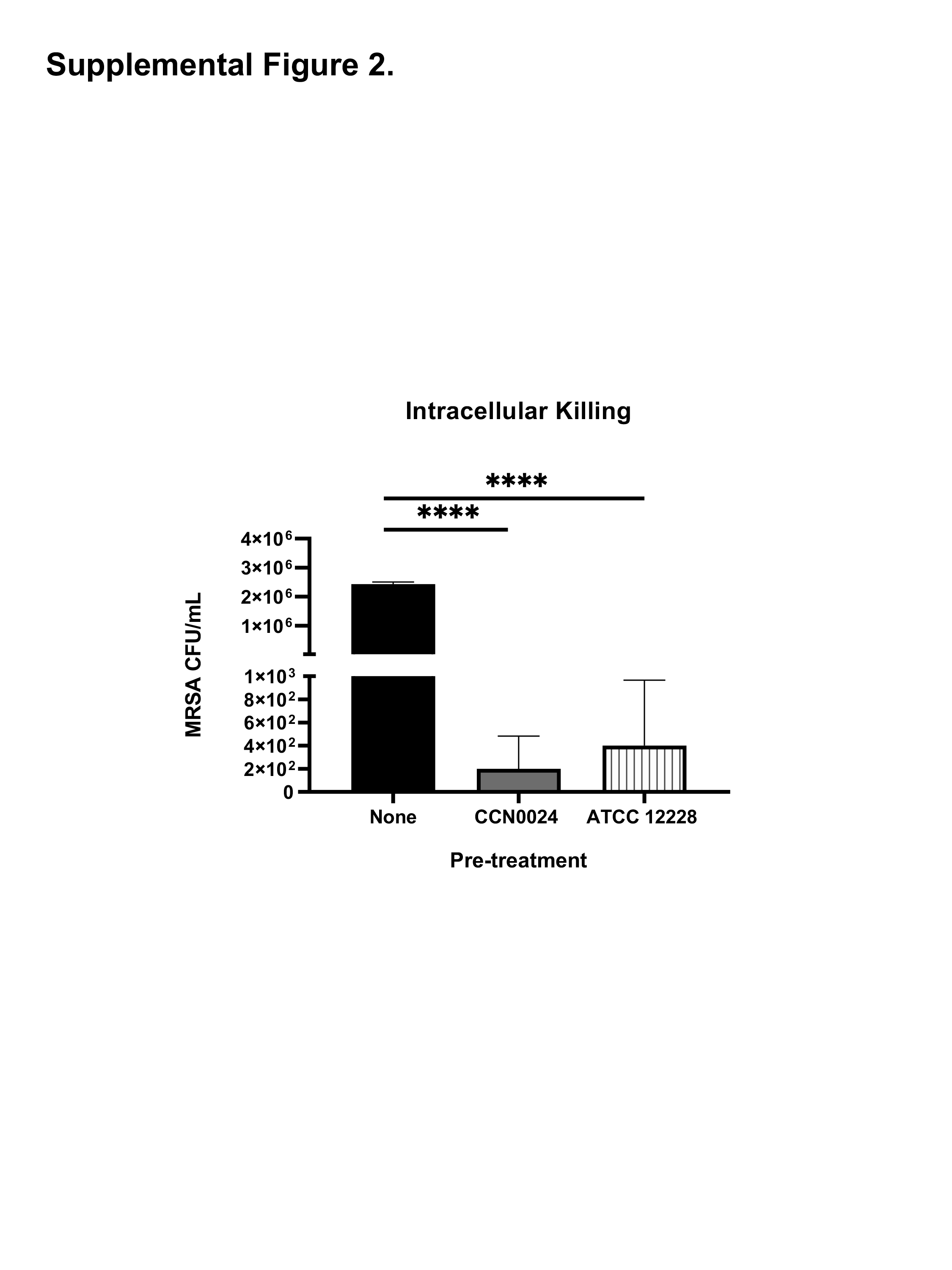

Supplement: FIGURE S2 — Pre-treatment of skin cells with S. epidermidis CCN0024 and ATCC 12228 strains limits intracellular MRSA survival. Single skin cells were exposed to single S. epidermidis strain at MOI 1:20 or media control for 24 h. After washing to remove S. epidermidis, cells were infected with MRSA (MOI 1:20) for 1 h to allow intracellular infection, and extracellular bacteria were subsequently removed by gentamicin treatment. Bar graph shows the number of intracellular MRSA (CFU/ml) upon hypotonic lysis of control and S. epidermidis pre-treated cells. ****p < 0.0001 as calculated using one-way ANOVA with Dunnett’s multiple comparisons test, which compared each S. epidermidis pre-treatment with non-pretreated control cells. [file Image_2.tif]
